# Blood Glucose Control Using a Novel Continuous Blood Glucose Monitor and Repetitive Intravenous Insulin Boluses: Exploiting Natural Insulin Pulsatility as a Principle for a Future Artificial Pancreas

**DOI:** 10.1155/2013/245152

**Published:** 2013-11-27

**Authors:** Nils K. Skjaervold, Dan Östling, Dag R. Hjelme, Olav Spigset, Oddveig Lyng, Petter Aadahl

**Affiliations:** ^1^Department of Circulation and Medical Imaging, Norwegian University of Science and Technology, MTFS, Postbox 8905, 7491 Trondheim, Norway; ^2^Department of Anesthesiology and Intensive Care Medicine, Trondheim University Hospital, Postbox 3250 Sluppen, 7006 Trondheim, Norway; ^3^Invivosense Norway Ltd., c/o NTNU Technology Transfer, Sem Saelands Vei 14, 7034 Trondheim, Norway; ^4^Department of Electronics and Telecommunications, Faculty of Information Technology, Mathematics and Electrical Engineering, Norwegian University of Science and Technology, 7491 Trondheim, Norway; ^5^Faculty of Technology, Sør-Trøndelag University College, Postbox 2320, 7004 Trondheim, Norway; ^6^Department of Laboratory Medicine, Children's and Women's Health, Norwegian University of Science and Technology, 7491 Trondheim, Norway; ^7^Department of Clinical Pharmacology, Trondheim University Hospital, Postbox 3250 Sluppen, 7006 Trondheim, Norway; ^8^Unit of Comparative Medicine, Norwegian University of Science and Technology, 7491 Trondheim, Norway

## Abstract

The aim of this study was to construct a glucose regulatory algorithm by employing the natural pulsatile pattern of insulin secretion and the oscillatory pattern of resting blood glucose levels and further to regulate the blood glucose level in diabetic pigs by this method. We developed a control algorithm based on repetitive intravenous bolus injections of insulin and combined this with an intravascular blood glucose monitor. Four anesthetized pigs were used in the study. The animals developed a mildly diabetic state from streptozotocin pretreatment. They were steadily brought within the blood glucose target range of 4.5–6.0 mmol/L in 21 to 121 min and kept within that range for 128 to 238 min (hypoglycemic values varied from 2.9 to 51.1 min). The study confirmed our hypotheses regarding the feasibility of this new principle for blood glucose control, and the algorithm was constantly improved during the study to produce the best results in the last animals. The main obstacles were the drift of the IvS-1 sensor and problems with the calibration procedure, which calls for an improvement in the sensor stability before this method can be applied fully in new studies in animals and humans.

## 1. Introduction

The development of an artificial endocrine pancreas (AEP), composed of a system of continuous blood glucose monitoring and automated insulin infusion, has been a long sought-after solution in diabetic care [1]. The intravascular approach to both glucose sensing and insulin delivery was abandoned in the late 80s, so current research is focused on subcutaneous glucose measurements and subcutaneous insulin administration [2]. However, the performance of these systems are still less than satisfactory, even though there have been some recent advances [3–5]. We think it is time to reexamine the intravascular approach as this calls for substantial benefits despite its invasiveness.

Recent research has demonstrated the pulsatile nature of pancreatic beta cells. A convincing body of evidence indicates that insulin is secreted in synchronized bursts from the entire pancreas into the portal blood stream [6–8]. Likewise, multiple studies in humans and animals have described the oscillatory nature of systemic levels of blood glucose and insulin [9–12]. Pulsatile pancreatic activity seems to be lost in advanced type 2 diabetes [13]. Pulsed intravenous insulin delivery has been shown to be more effective in lowering BGL compared to equal doses of continuously infused insulin [14, 15], and the pulsatile nature of endogenous insulin secretion has been mimicked for therapeutic reasons as pulsatile intravenous insulin therapy. Compared to standard therapy, pulsed therapy has shown better metabolic control, less end-organ damage, and restoration of normal pulsatile pancreatic function in type 2 diabetes [16–18].

**Figure 1:**
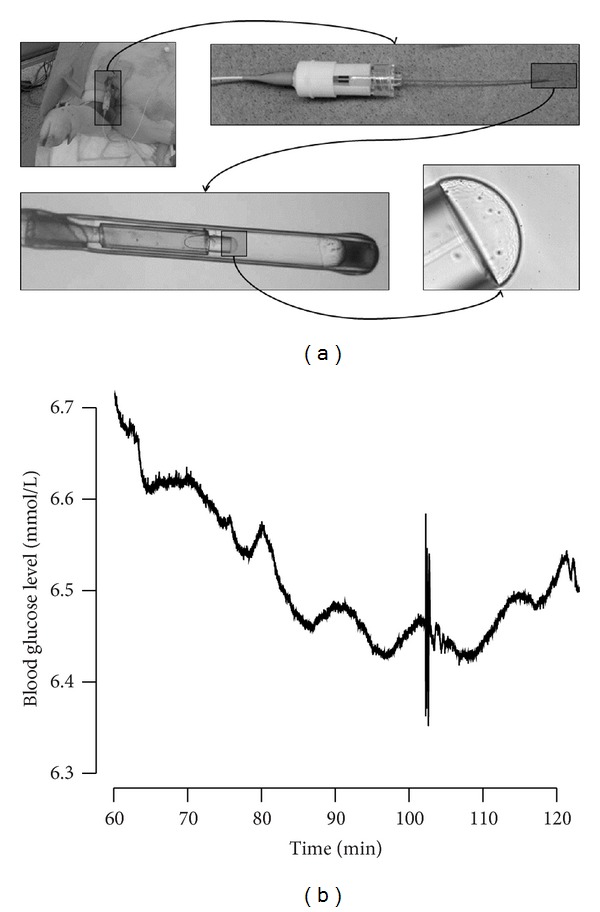
(a) Pictures that show the hydrogel part of the IvS-1 continuous blood glucose monitor prototype at increasing magnification; (b) a classic example of oscillating blood glucose levels with a periodicity of approximately 10 minutes and an amplitude of approximately 0.05 mmol/L (from animal 2, which was only mildly affected by the streptozotocin pretreatment; measurements from 60 to 120 minutes in the actual experiment after IvS-1 stabilization but before the first insulin bolus; artifact seen at 103 minutes).

In this study, we used a novel intravascular continuous glucose sensor, the IvS-1 (Invivosense, Trondheim, Norway) [19] (Figure 1(a)). Our group has tested the IvS-1 in preclinical *in vivo* studies in pigs and found that it demonstrated high accuracy and a rapid response time [20]. The sensor was originally developed to meet the need for better control of blood glucose levels (BGL) in patients in intensive care units. However, when we discovered the potential of our technology, we started to work towards the development of an AEP. We realized that the sensor was able to instantaneously detect very small changes in BGL, and we found small oscillations in BGL with a period of approximately 10 minutes (Figure 1(b)).

When we first started to infuse insulin intravenously to regulate BGL, we found that the time from start of the infusion until a new steady state BGL was reached to be two hours or longer, which is very long. This means that it would take several hours to adjust any insulin infusion to the correct rate to achieve an appropriate and stable BGL. The insulin resistance in a single individual is constantly changing [21], and some authors even suggest that the regulatory system includes deterministic chaotic components that would render it impossible to foresee the effect of insulin on BGL during a given time using ordinary linear methods [22]. A control system based on continuous infusions will therefore always be “running to catch up” and will have great difficulties in lowering a patient's BGL to a sufficient degree without risking hypoglycemia. The use of *subcutaneous* sensors and infusions would increase the time delay and complicate the situation even more.

Given this background, we conducted a series of experiments to characterize the effects of intravenous bolus injections of insulin (IB) in a previous study [23]. Here, we found the time lag from an IB until a first observed decrease in BGL becomes four to six minutes. The maximum rate of decrease in BGL occurred shortly thereafter, and a nadir was reached 15–20 minutes after the IB. These time intervals seemed to be rather dose independent—as long as the IB dose was sufficient to yield any change in BGL at all.

Based on these observations, we hypothesized that it should be possible to construct an AEP using the biological pancreas as a model. The IvS-1 would detect early changes in BGL, and the system should respond instantaneously with adjusted repetitive IBs. We had to construct a novel algorithm with the target of establishing and maintaining a normal fasting BGL, defined as 4.5–6.0 mmol/L. BGL regulation had to be a two-stage process, with the first step to establish glycemic control in a hyperglycemic subject by rapidly bringing BGL to the desired level and thereafter to maintain the desired glucose level over time.

## 2. Materials and Methods

### 2.1. The Insulin Algorithm and Administration

We identified the following key elements that had to be incorporated in the algorithm.

1. The overall goal was to bring BGL to within a predefined range of 4.5–6.0 mmol/L and to keep it in this range, including small oscillations around the middle value in this range.
2. IB was administered whenever needed in accordance with the continuous BGL readings; adjusting the IB doses was kept simple with only three alternatives according to the previously administered IB: the same dose as the previous one, half the previous dose, or twice the previous dose.
3. Any decrease in BGL should be observable within five minutes after an IB; otherwise, a new IB should be administered.
4. Blood glucose control should be *established* by a rapid decrease in BGL from hyperglycemic levels with a series of consecutive IBs. The BGL should be lowered ≥1 mmol/L for each IB injected.
5. As soon as the BGL dropped below 6.0 mmol/L, blood glucose control should be *maintained* by meticulously timed IBs to allow small fluctuations in the blood glucose curve.

**Figure 2:**
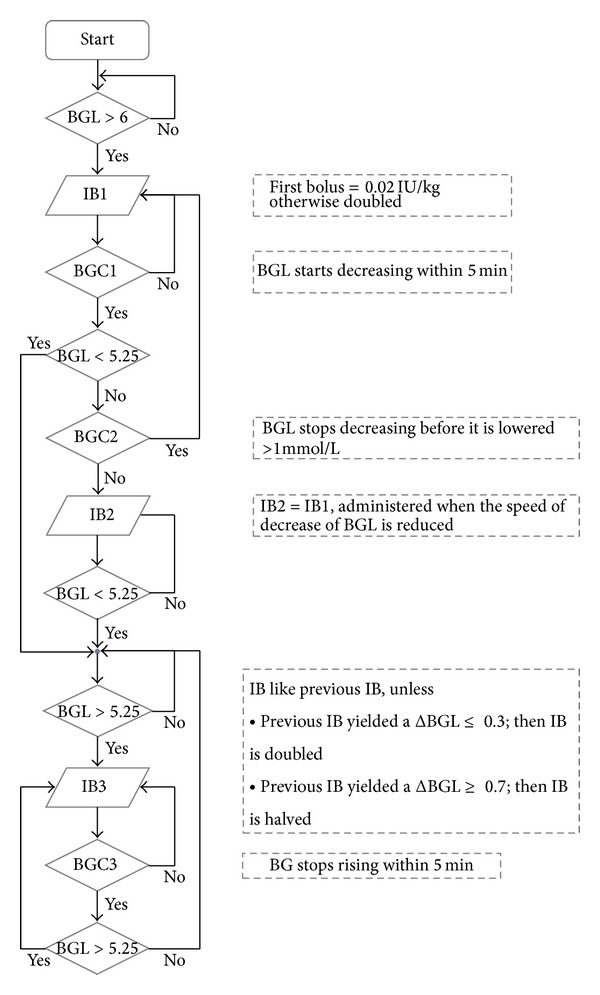
The current insulin algorithm; BGL: blood glucose level with the value in mmol/L; IB1–3: insulin bolus 1 to 3; BGC1–3 blood glucose control 1–3; ΔBGL the total amplitude in blood glucose level between two consecutive insulin boluses.

The insulin algorithm was constructed as a flowchart based on simple IF-THEN decisions, and the details of the algorithm evolved as the study went on. The current version of the algorithm is shown in Figure 2.

Human recombinant insulin 0.1 IU/mL (Actrapid, NovoNordisk, Bagsværd, Denmark) was used in the study. The drug was manually administered from a syringe pump (using the bolus function) (Alaris CC Plus, CareFusion, San Diego, CA, USA) in accordance with the insulin algorithm.

### 2.2. The Animal Model

The study was approved by the Norwegian State Commission for Animal Experimentation. Forty-eight hours prior to the main experiments, we induced diabetes in healthy pigs by destroying their pancreatic beta cells using the cytotoxic agent streptozotocin 200 mg/kg i.v. (Zanosar, Teva Parenteral Medicines, Irvine, CA, USA) [24, 25]. On the day of the experiment, the animals were put under general anesthesia, an arterial line was established for monitoring and blood samples, central venous access was obtained, and the animals were instrumented with two IvS-1 sensors, one in each femoral artery. Animal handling, anesthesia, and surgical intervention have been described in previous studies [20, 23].

### 2.3. IvS-1 Calibration

In order to achieve real-time continuous BGL output, the IvS-1 software had to be updated and a preinsertion *in vitro* calibration procedure had to be determined. We applied a calibration procedure by exposing the sensor to buffer solutions with glucose concentrations of 0.0, 2.0, and 10.0 mmol/L. A nonlinear least square algorithm was used to compute the two calibration parameters describing the nonlinear calibration function. After insertion, the IvS-1-signal quickly stabilized. The time series from the IvS-1 was calibrated using the computed calibration parameters and a one-point calibration method to set the off-set parameter by adjusting the IvS-1 level with the blood glucose level achieved from a simultaneously drawn blood-sample analyzed on a bed-side Radiometer ABL 720 blood-gas analyzer (Radiometer, Brønshøy, Denmark). Throughout the study, several *in vivo* calibration procedures were performed to adjust for the inherent drift in the sensor. To ensure we had redundancy, we instrumented each animal with two IvS-1 sensors hardwired to the same monitor. After the first *in vivo* calibration we chose whichever one had the most stable signal and used data from this sensor for the rest of the experiment.

### 2.4. Data Handling, Analysis, and Statistics

After the experiment, the IvS-1 data was retrieved by using data from the repetitive blood samples as calibration parameters. To transform the interferometric length measurement data into glucose concentration data, we used the nonlinear two-parameter calibration function described previously. We compensated for baseline drift by using a fixed baseline drift rate and compensated for pH interference by using the pH values from the blood-gas analyzer to compute pH corrected calibration parameters. The pH dependence of the calibration parameters was found from a set of *in vitro* experiments.

## 3. Results

**Figure 3:**
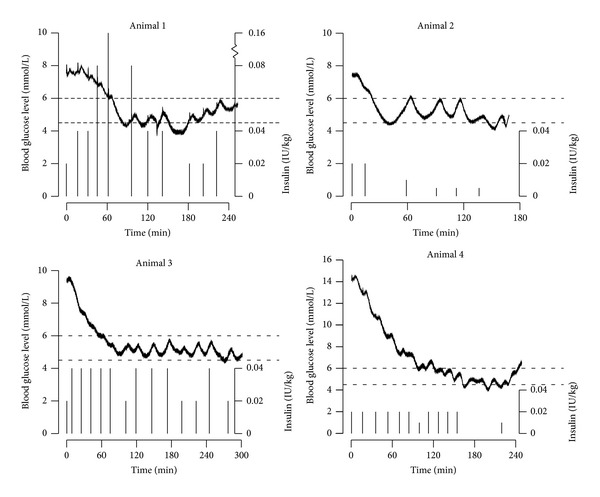
Blood glucose control in four animals; the curves depict the blood glucose level, the histograms depict the insulin boluses, and the horizontal dashed lines depict the ideal blood glucose target interval of 4.5 to 6.0 mmol/L.

This paper presents data from four animals, with the details shown in Figure 3 and Table 1. The glucose values presented are those after the calibration procedures described in the previous paragraphs. The effect of the streptozotocin pretreatment varied between animals; at the time of the first insulin bolus the starting BGL value (BGL_0_) ranged from 7.46 to 14.06 mmol/L. We were able to establish glycemic control by bringing the BGL below 6.0 mmol/L (*T*_est_) in 21 to 121 minutes, with the longest time in animals with a high BGL_0_ value. In animals 2–4, the rate of decrease (RD) from the first insulin bolus until the 6.0 mmol/L limit was reached was relatively high, from 0.064 to 0.077 (mmol/L)/min. (As a comparison, we found the maximum RD to be approximately 0.1 (mmol/L)/min after a single bolus dose of insulin in our earlier experiments [23].) After reaching the target range, the animals were kept under glycemic control (*T*_ctrl_) from 128 to 238 minutes. The total time with BGL values below the lower limit of the range, that is, 4.5 mmol/L (*T*_low_), varied from 3 to 51 min in the four animals. The lowest BGL measured in the four animals (BGL_low_) varied from 3.81 to 4.44 mmol/L.

## 4. Discussion

The intuitive interpretation of Figure 3 is that the fundamental principles of this AEP model work. BGL was rapidly and safely brought within the predefined range and kept within the range during the study time. We tried to administer IB as best we could according to the algorithm; however, small details in the control algorithm had to be updated and changed as the experiments went on. These were mainly the timing of the boluses as the BGL tends to rise quite fast, and it is important not to delay the IB as it takes approximately 4 minutes from bolus given until a change in BGL starts to occur. We found that the animals behaved very differently in terms of their insulin needs. However, the time lag between IB and effect was predictable and in accordance with our previous research.

The amplitude in BGL variations during established control was somewhat larger than desired. We believe that the amplitude can be substantially decreased by further improvement in blood glucose sensor stability and improvement in the insulin regulatory algorithm. As explained in the introduction, actual pancreatic beta cells oscillate with a fixed time interval of approximately five minutes, whereas the amount of insulin released with each pulse is constantly changing. As the effect of a single IB starts to lower BGL in four to six minutes and the nadir BGL values are reached after approximately 15 minutes, consecutive insulin bursts from the pancreas with an interval of five minutes will yield a carryover effect where several bursts interfere with BGL at a given time. We found this regulation system to be too complicated to model at this stage and therefore constructed a simplified system where the effect of each IB was followed throughout its effect period before a new IB was administered. In the future, a model able to correct for, and take advantage of, this carryover effect might be constructed, either through the use of fixed interval IBs and/or IBs combined with a small continuous infusion of insulin. Another limitation of our AEP is that insulin is infused into the systemic circulation, whereas the pancreas secretes the insulin into the portal vein. This means that in our model (as in all other available insulin infusion regimes) the liver receives relatively small amount of insulin compared to the rest of the body.

Current AEP models are based on subcutaneous glucose measurement and subcutaneous insulin administration. The insulin infusion algorithms are based on what is called model predictive control, where the pharmacokinetic properties of insulin are modeled in a series of equations that are used to calculate the correct insulin dose at a given time [26, 27]. Several studies have shown the feasibility of such systems in controlling BGL overnight in diabetic subjects. However, it is very challenging to calculate the correct insulin dose when the subject is eating, exercising, or ill, and a fully automatic AEP thus has yet to be constructed [28, 29]. We believe a more empirical system like ours could tackle such obstacles, as it allows for the correct insulin dose to be calculated and timed continuously based on the effect of the previous IB. In this system, it is very unlikely that BGL would drift into hypoglycemic levels or that it would rise into gross hyperglycemia.

The main challenge during the study was that the IvS-1 was not optimally calibrated at all times, which led to small errors in the real-time BGL display. We therefore to some extent had to rely on a combination of the IvS-1 output and the blood samples to estimate the correct real-time BGL in order to use the insulin algorithm properly. The observed periods of BGL below the range's lower limit were caused by a discrepancy between the observed real-time BGL and the correct BGL calculated after the experiment (the reported BGL). As such, the major limitation of the current technology is a background swelling of the glucose-sensing hydrogel, which resulted in a drift in the IvS-1 output. Our current work is focused on enhancing the hydrogel.

There will always be some doubt as to whether results from animal studies are valid in a human population; on the other hand, the model makes it possible to manipulate BGL and to instrument study subjects without fear of any iatrogenic damage to healthy volunteers or patients. The IvS-1 probes used are all handcrafted and the setup of the animal experiment is complicated, which is why the number of animals used in the study is low. However, all of the animals studied (both the final four as well as animals in our earlier studies) displayed the same behavior. The main point of this paper has been to illustrate the physiological principle of using nature's own regulatory system in an artificial control system, without fine tuning the details.

## 5. Conclusions

We conclude that the use of real-time accurate intravasal glucose monitoring in combination with repetitive bolus injections of insulin, administered to mimic the natural pulsatility of endogenous insulin, is a promising method for a future artificial pancreas.

## Figures and Tables

**Table 1:** The seven outcome variables from the four animals; BGL_0_: the blood glucose level at the time of the first insulin bolus; BGL_low_: the lowest blood glucose level recording during the experiment; *T*_est_: time to establish glycemic control from the first insulin bolus until the blood glucose level went below 6.0 mmol/L; RD: the rate of blood glucose level decrease during *T*_est_; *T*_ctrl_: time with glycemic control from blood glucose level went below 6.0 mmol/L until end of experiment; *T*_low_: total time with blood glucose levels below 4.5 mmol/L during *T*_ctrl_; *T*_range_: percentage time with BGL in the correct range between 4.5 and 6.0 mmol/L during *T*_ctrl_.

Animal	BGL_0_ (mmol/L)	BGL_low_ (mmol/L)	*T*_est_ (min)	RD ((mmol/L)/min)	*T*_ctrl_ (min)	*T*_low_ (min)	*T*_range_ (%)
1	7.53	3.81	68	0.025	186	51	73
2	7.46	4.14	21	0.073	146	20	86
3	9.33	4.44	63	0.064	238	3	99
4	14.06	4.04	121	0.077	128	22	83
